# Inter- vs. Intramolecular Hydrogen Bond Patterns and Proton Dynamics in Nitrophthalic Acid Associates

**DOI:** 10.3390/molecules25204720

**Published:** 2020-10-14

**Authors:** Kinga Jóźwiak, Aneta Jezierska, Jarosław J. Panek, Eugene A. Goremychkin, Peter M. Tolstoy, Ilya G. Shenderovich, Aleksander Filarowski

**Affiliations:** 1 Faculty of Chemistry, University of Wrocław 14 F. Joliot-Curie str., 50-383 Wrocław, Poland; kin.joz@o2.pl (K.J.); aneta.jezierska@chem.uni.wroc.pl (A.J.); jaroslaw.panek@chem.uni.wroc.pl (J.J.P.); 2 Frank Laboratory of Neutron Physics, Joint Institute for Nuclear Research 6 F. Joliot-Curie str., 141980 Dubna, Russia; goremychkin@jinr.ru; 3 Institute of Chemistry, St. Petersburg State University, Universitetskij pr. 26, 198504 St. Petersburg, Russia; peter.tolstoy@spbu.ru; 4 Institute of Organic Chemistry, University of Regensburg, Universitaetstrasse 31, 93053 Regensburg, Germany

**Keywords:** proton dynamics, carboxyl group, CPMD, DFT, IINS, IR, Raman, NMR

## Abstract

Noncovalent interactions are among the main tools of molecular engineering. Rational molecular design requires knowledge about a result of interplay between given structural moieties within a given phase state. We herein report a study of intra- and intermolecular interactions of 3-nitrophthalic and 4-nitrophthalic acids in the gas, liquid, and solid phases. A combination of the Infrared, Raman, Nuclear Magnetic Resonance, and Incoherent Inelastic Neutron Scattering spectroscopies and the Car–Parrinello Molecular Dynamics and Density Functional Theory calculations was used. This integrated approach made it possible to assess the balance of repulsive and attractive intramolecular interactions between adjacent carboxyl groups as well as to study the dependence of this balance on steric confinement and the effect of this balance on intermolecular interactions of the carboxyl groups.

## 1. Introduction

Hydrogen bonding (H-bonding) and steric effects are important tools of molecular engineering. Under certain conditions, their interplay can stabilize species that otherwise exhibit high chemical reactivity [1,2,3,4]. The structural complexity increases when there are either other noncovalent interactions or competing H-bonds. The former is critically important in solids [5,6,7,8,9], at confined geometries [10,11,12], and in aqueous solutions [13,14,15,16]. The latter is characteristic for P=O moiety [17,18], specially designed organic molecules [19,20], but most of all for biomolecules [21,22]. The adjustments of bridging proton positions in H-bonds act as one of the mechanisms governing the chemical properties of macromolecules [23,24,25] and biosystems [26,27]. Changes of weak specific interactions such as H-bonds can evoke a reorganization on the macroscopic scale. Therefore, many-sided elaborate studies of the conformational phenomena are essential not only for fundamental understanding of H-bond nature but also for a number of practical applications, such as design of materials with the required physicochemical properties [28,29,30].

The wide variety of effects associated with a competition between intra- and intermolecular H-bonding can be illustrated with salicylic acid. In the simplest case of salicylic acid crystals, the carboxyl groups of the molecules form dimers while their hydroxyl groups form intramolecular H-bonds [31,32]. This structure remains qualitatively valid in an aprotic solution when the dimer is deprotonated [33]. In contrast, when the number of competing interactions increases, the co-crystals of salicylic acid exhibit polymorphism and different solubility [32,34,35]. These changes are critically important for pharmaceutical applications. Besides that, the intramolecular H-bond in salicylic acid derivatives can be controlled through intramolecular steric effects. In the crystalline salicylic acid, the O^…^O distances of this H-bond are about 2.62 Å [31,32]. In 2-hydroxy-3-nitrobenzoic acid, 6-(cyclohexylmethyl)salicylic acid, and 6-(2-cyclohexylethyl)salicylic acid they are only 2.55, 2.54, and 2.52 Å, respectively [32,36]. Is this a general trend that can be expected for other molecules’ structures?

**Figure 1:**
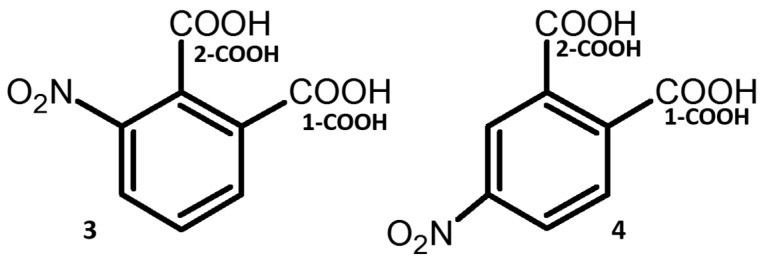
Chemical structures of 3-nitrophthalic (**3**) and 4-nitrophthalic (**4**) acids.

This paper presents the conformational studies of 3- and 4-nitrophthalic acids (**3** and **4**, Figure 1). These compounds are characterized by the presence of strong intermolecular and intramolecular H-bonds in co-crystals with various organic compounds [37,38,39,40,41]. These bonds might mutually convert one into another in compounds with adjacent carboxyl groups under impact of external factors. The first papers about dimeric formation by carboxyl group were published by Pfeiffer et al. in 1910 [42,43,44]. The carboxylic acid dimer units (2 × (COOH)) have still attracted attention for researchers involved in H-bonding studies [45,46,47,48,49,50,51,52,53,54,55]. In References [56,57,58], authors show a strong effect of H-bonds on the conformational state of compounds. The domination of *cis* conformation of carboxyl group, so-called *Z*-effect, has been elucidated by Lyssenko et al. [59]. Recently, two polymorphic forms of cinchromeronic acid (the derivative of phthalic acid) have been discovered and studied [60]. It has been shown that the polymorphic forms are caused by the proton transfer and reorientation of the carboxyl groups. Computational studies of H-bonds and stable conformers are important for the development of the conformational polymorphism of the molecular complexes such as benzoic acid with pyridine [61]. Moreover, H-bonded networks of phthalic acids can be used as ligands for metal-organic aggregates [62,63].

The main aim of this study was to characterize intramolecular interactions between adjacent carboxyl groups in the presence and absence of intramolecular steric effects and the effect of all these interactions on intermolecular interactions of these carboxyl groups. The nitro substitution was chosen because this moiety is rigid, relatively small, and causes considerable steric strain. Besides static density-functional theory (DFT) computations, this study covers simulations performed using the Car–Parrinello molecular dynamics (CPMD) approach, which supports NMR (Nuclear Magnetic Resonance), IR (infrared), Raman, and IINS (Incoherent Inelastic Neutron Scattering) experimental measurements with the employment of a neutron radiation source.

The outline of the manuscript is as follows. Firstly, the conformational analysis on the basis of static DFT calculations is presented. Next, the proton and functional groups’ dynamics were studied by DFT and CPMD calculations. The following part delves into the investigations of conformational equilibrium in the solutions accomplished by NMR spectroscopy as well as IR, Raman, and IINS studies of the compounds in the solid state. Additionally, the spectral analysis on the basis of the experimental and computational results by means of H/D isotopic substitution was performed. The concluding remarks are given in the last section.

## 2. Results and Discussion

### 2.1. DFT Study of H-Bond, Nitro, and Carboxyl Groups’ Dynamics of Nitrophthalic Acids

**Figure 2:**
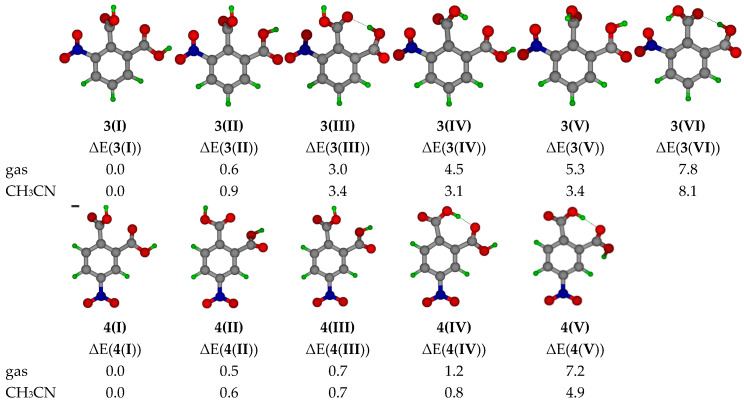
Conformers of monomeric **3** (upper row) and **4** (bottom row) and their relative energies (Δ*E* = *E*_min_(conformer) – *E*_i_(conformer), kcal/mol) obtained at the B3LYP/6-311+G(d,p) level of theory for the gas phase and in acetonitrile (CH_3_CN). *E*_min_(conformer) stands for the energy of 3(I) or 4(I). *E*_i_(conformer) stands for the energy of the conformer under consideration.

The quantum-mechanical calculations were accomplished at the B3LYP/6-311+G(d,p) level of theory for the detection of the most stable conformer of monomeric **3** and **4**. These calculations show that the most stable conformer does not contain the intramolecular H-bond (Figure 2). Generally, intramolecular H-bonds can significantly decrease the energy of isolated molecules [64]. However, conformers **3(III)**, **3(IV), 3(VI), 4(IV),** and **4(V)** with the intramolecular H-bond feature significant steric tensions between the carboxyl groups that increases further if the nitro group is nearby. In consequence, the energies of conformers **3(III)** and **4(IV)** are higher as compared to **3(I)** and **4(I)**.

Using the knowledge of the monomer’s conformations, the calculations and analysis of the possible structures of hydrogen-bonded dimers were performed (labelled **D3** and **D4** in Figure S1). The most stable conformation of the dimers was obtained when the molecules were arranged orthogonally (**D3(I)**, **D3(II)**, **D4(I),** and **D4(II)**, Figure S1). However, the planar orientations of the molecules caused only a small increase in energy (**D3(III)**, **D3(IV)**, and **D4(V)**. Indeed, in the crystal of **3,** one carboxyl group of each molecule formed a dimer with the planar orientations of the rings while the other carboxyl group formed a hydrogen-bonded molecular chain between such dimers [37]. Structures in which the second carboxyl group was oriented orthogonally to the intermolecular H-bonded group were energetically beneficial. This result was conditioned by a smaller steric repulsion between carboxyl groups (and the nitro group in case of **3**). Thus, the formation of intramolecular H-bonds in the dimers was unfavorable. Oligomers **D3(IX)** and **D4(VIII)**, in which molecules did not form carboxyl group dimers, exhibited higher energies (Figure S1). However, the energy increase was quite moderate, especially for compound **4**. Moreover, **D3(X)** and **D4(VII)** possessed one intramolecular H-bond each.

For the assessment of dynamic effects associated with the rotations of carboxyl and nitro groups, the corresponding potential energy profiles were calculated at the B3LYP/6-311+G(d,p) level of theory for the monomers of both acids. The DFT calculations of the rotation of the nitro groups (gradual increase of the torsional angles C2C3NO5 in **3** and C3C4NO5 in **4** (Figure S2)) revealed the similarity of the rotational energy barriers for both compounds: 4.8 and 5.8 kcal/mol for **3** and **4**, respectively (Figure S3). These barriers resulted from the disruption of the π-electronic coupling between the nitro group and the benzene ring, which caused energetically disadvantageous configurations at CCNO ≅ 90° (Figure S3). For **3**, one can also observe a small barrier at C2C3NO5 ≅ 180°, caused by the repulsion between the nitro and carboxyl groups (Figure S3). It is noteworthy that the rotation of either nitro or carboxyl group evoked the simultaneous rotation of the neighboring functional groups and, therefore, it led to moderately high energy barriers.

**Figure 3:**
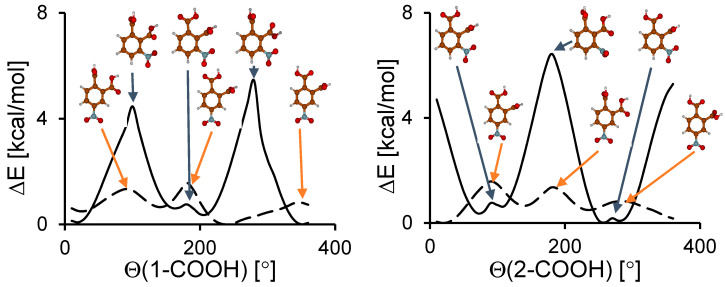
Calculated potential energy curves for the carboxyl group rotation in **3** (solid line) and **4** (dashed line).

In contrast to the nitro group rotation, the calculations showed a significant difference between the energy barrier heights for the rotation of the carboxyl groups in **3** and **4** (Figure 3). For **3**, these barriers were 5.5–6.5 kcal/mol, which was 4–5 kcal/mol higher than for **4**. This difference resulted from a strong steric effect between three functional groups in **3**. The steric squeezing between carboxyl groups in **4** was weaker than in **3** because the nitro group was in the *meta* position. The energy barriers for the nitro and carboxyl groups’ rotation were not very high. Thus, for these compounds a significant dynamics of all functional groups can be expected.

**Figure 4:**
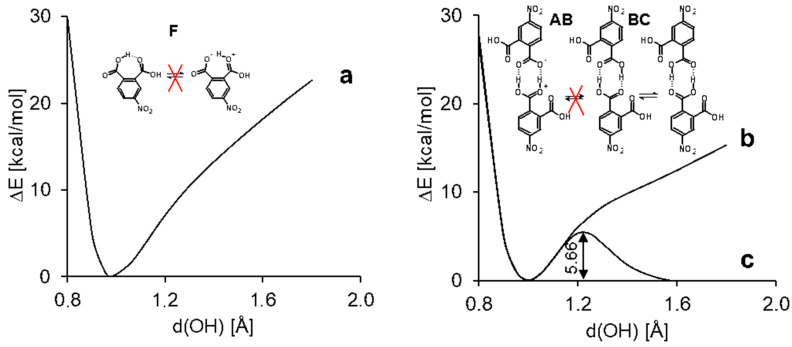
The potential energy profile for a gradual displacement of one proton within the H-bond in the **4(II)** monomer (**a**) and the **D4(I)** dimer (**b** and **c**) calculated in the PCM approximation in acetonitrile. The curves **a** and **c** represent a case when all other structural parameters are optimized. The curve **b** represents a case when the position of the adjacent bridged proton is fixed.

**Figure 5:**
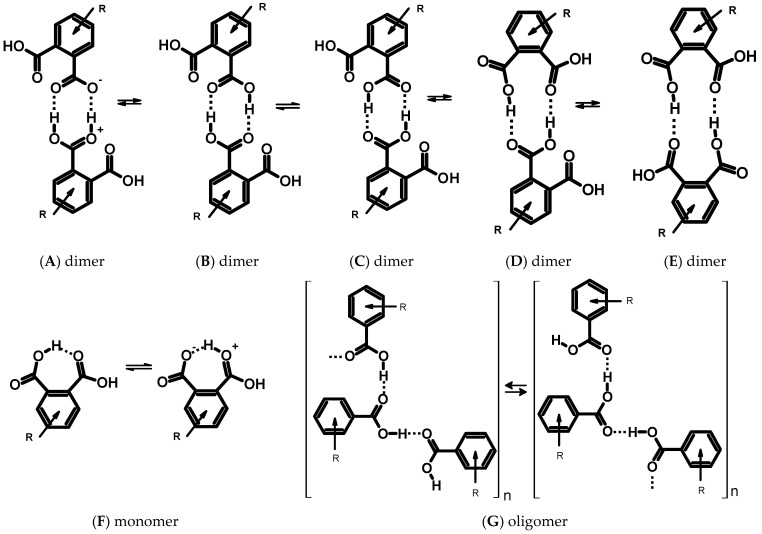
Schemes of the prototropic equilibria for the carboxyl aryl derivatives and their intermolecular complexes.

In order to study the H-bond dynamics, we calculated potential energy profiles for proton transfer in intramolecular H-bonds in **3(III)**, **4(IV)**, **D3(III)**, and **D4(I)** in the gas phase and taking into account the effect of a polar solvent (CH_3_CN) using the polarizable continuum model (PCM) approach (Figure 4 and Figure S4a,b). The O-H distance in one of the carboxyl groups was gradually elongated while other structural parameters were optimized for each step. The profile of the curves and its numerical values were similar for monomers and dimers. The calculations of the potential energy curves for the intramolecular proton transfer in the monomeric species showed no second minimum in the range of O⋯H distances 1.4–1.7 Å (Figure 4, curve **a**). According to the earlier presented analysis [65], which rests upon the experimental and computational data, this result proves the absence of the proton transfer within the intramolecular hydrogen bond, i.e., the absence of a tautomeric equilibrium (Figure 5F). In turn, the intermolecular transfer of one proton within the intermolecular hydrogen bond in dimers of compound **3** induced a transfer of the second proton in the adjacent intermolecular hydrogen bond (Figure 4, curve **c**). The calculated potential energy curves for dimers **D3(III)** and **D4(I)** turned to be double-well. The energy required for this concerted double proton transfer was about 6.3 kcal/mol for the gas phase (Figure S4b). The use of the PCM approximation for acetonitrile reduced the barrier down to 5.7 kcal/mol (Figure 4). This fact supports the possibility to observe the tautomeric equilibrium with double proton transfer (Figure 5B,C) in an experiment. Taking into account that the PCM approximation strongly underestimates the effect of polar media on hydrogen-bonded systems [66,67,68,69], one can expect a fast, concerted proton transfer in phthalic acid dimers in polar solvents. Previously, a double proton transfer in carboxylic acid dimers was experimentally detected in low-temperature NMR spectra (110 K, CDF_3_/CDF_2_Cl mixture as solvent) as a triplet splitting of the bridging proton signal for ^13^C-labelled acetic acid due to ^2^*J*(C,H) spin-spin coupling [70]. Though dimers **D3(I)**–**D3(VII)** are the most stable forms, the formation of oligomers (structure **G**, Figure 5) and complexes of other types (**D** and **E** dimers, Figure 5) is also possible. This fact is also supported by the crystallographic and spectroscopic studies [71,72,73,74].

To explore the possibility of a single proton transfer in the dimers (equilibrium **BA**, Figure 5), the calculation was performed at a constant O-H distance of the adjacent hydrogen bond. There was no local minimum on the potential energy curve (Figure 4, curve **b**). Therefore, the formation of a zwitterionic complex (Figure 5, structure **A**) was disadvantageous and there was a poor chance to observe the equilibrium **BA** experimentally (Figure 5). Nevertheless, the profile for the single proton transfer in the dimer was more shallow than that in the monomer.

The calculated H-bond energies (Δ*E*(HB) ≈ *E*_min_(non-HB) − *E*_i_(HB) [75]) in the studied dimeric complexes were smaller than 7 kcal/mol per H-bond. The estimated values of the energies calculated for the dimers of **3** and **4** correlated well with the energies reported for similar systems. For example, according to the experimental temperature-dependent attenuated total reflection (ATR) IR studies of ibuprofen by Ludwig et al. [76], the enthalpy of the transition between doubly H-bonded cyclic dimers to singly H-bonded linear dimers is equal to −5.07 kcal/mol. The binding energy of a *p*-biphthalate dimer obtained at the B3LYP/6-31+G* approximation is about 12.4 kcal/mol (6.2 kcal/mol per one hydrogen bond) [77]. Such H-bonds are characterized as weak ones. However, the studied dimers exhibited an easy double proton transfer. Such phenomenon is typical for Strong Short H-Bonds (SSHB) [78]. This observation can be rationalized as follows. An elongation of the OH distance results in an increase of the electron density on the adjacent oxygen of the same carboxyl group, thereby it strengthens the basicity of this oxygen. When the OH bond length is ca. 1.25 Å, the basicity of the adjacent oxygen becomes sufficient to evoke a spontaneous transfer of the adjacent proton from the opposite carboxyl group. A further elongation of the OH bond brings about a moderate decrease of the dimer energy, thus creating a double-well potential. Following Gilli’s terminology [79], this phenomenon can be called charge flow-assisted hydrogen bond.

### 2.2. Dynamics of Hydrogen Bonding within the Framework of Molecular Dynamics

Molecular dynamics (MD) schemes, which reproduce time evolution of the studied systems, are useful in the investigations of multi-dimensional and complex phenomena [80,81,82]. In the studied case of phthalic acid derivatives, it was necessary to use the Car–Parrinello MD scheme (CPMD), which is based on the DFT framework and is able to reproduce H-bond properties [83,84,85,86,87,88]. This section describes how these CPMD simulations illustrate the impact of H-bond strength on the molecular metric parameters.

Table 1 presents statistical data (averages and standard deviations) for the CPMD production runs. After the thermostatted equilibration phase, the data collection without thermostats lasted 24 ps, and only the last 20 ps were taken as the production runs in order to allow the molecules to relax after thermostatting. It was interesting to see that the intramolecular H-bond in **4** was much stronger than in **3**, but the intermolecular bridges of the dimers were of almost the same strength. While the mean and standard deviations of the donor-acceptor distance were lower for a stronger bonding, the opposite was true for the donor-proton bond length. This was a result of increased delocalization of the proton in the stronger bridge, whereas the dynamics of the H-bridge were weaker. The donor-acceptor distances listed in Table 1 indicate that the intermolecular H-bonds in the dimer of **3** were stronger and more delocalized than the intramolecular one in the monomeric **3**. An opposite phenomenon was observed for **4**. This discrepancy can be explained by the difference in the geometry of the structures. For the dimers of **3** and **4**, the geometry of the H-bridges was planar (COH^…^O torsional angle ~0°) and linear (OHO angle ~179°), while, for the monomers, the geometry was neither planar nor linear (COH^…^O and OHO angles were ~64/50° and ~150/160° for **3**/**4**, respectively, Table 1). These deviations from the planarity were caused by a strong electrostatic repulsion between oxygen atoms of the intramolecular H-bonds in the monomers. Moreover, for the monomer of **3**, the phenomenon of non-coplanarity was enhanced by a strong steric repulsion from the nitro group, which led to an additional weakening of the intramolecular H-bond.

**Figure 6:**
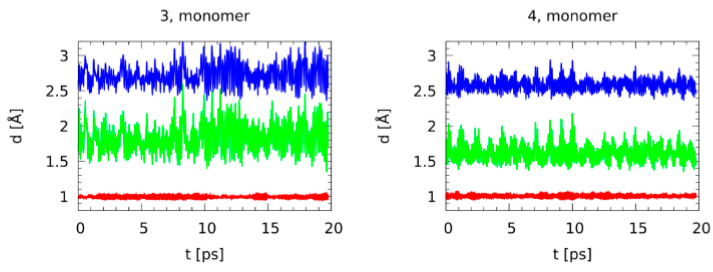
Time evolution of the H-bridge metric parameters. The CPMD gas phase simulations of the monomeric **3** and **4**. Red: Donor-proton distance, green: Proton-acceptor distance, blue: Donor-acceptor distance.

Additional insight was provided by the time evolution of the bridge distances, depicted in Figure 6 for the monomers and Figure S5 for the dimers. It was striking that even if the monomer of **4** had the shortest donor-acceptor distance among the studied systems, there were no indications of the proton entering the acceptor side. On the other hand, the intermolecular cyclic dimers of **3** and **4** were typical for carboxylic acids. For **4**, there were numerous instances of the bridge proton being located almost in the middle of the bridge, while for **3** there were just two such cases and one of them was a concerted transfer (occurring at the same time in both bridges). Such synchronicity was less obvious for **4**. This delocalization of the protons in the cyclic dimer of **4** showed that the H-bonding in **4** was stronger than in **3** for both the monomer and the dimer.

It is worth to note that the H-bond in the monomer of **3** was characterized by the greatest dynamics, due to its non-planar structure and, as a consequence, a significant deformation component.

### 2.3. NMR Studies of Nitrophthalic Acids

NMR study of H-bonding in solution is challenging due to the short lifetime of H-bonded complexes of low molecular weight. Generally, only a single NMR line is observed for all mobile protons, which represent an average over different, fast interconverting hydrogen-bonded complexes. This problem can be solved using a low-freezing solvent [89]. In this solvent complex, hydrogen-bonded systems can be characterized in great detail [90,91,92]. However, such experiments are not without their problems. A simplified qualitative analysis is possible when the mole fractions and the individual chemical shifts of different H-bonded complexes are known.

**Figure 7:**
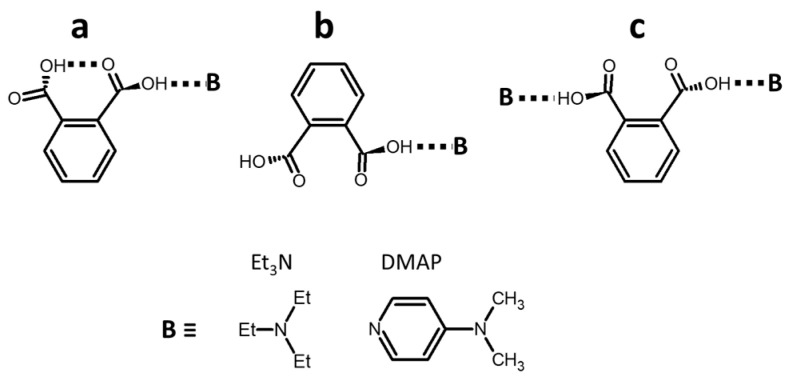
Possible scenarios of phthalic acid interaction with bases in nonpolar solution: (**a**) One intra- and one intermolecular H-bond, (**b**) single intermolecular H-bond, and (**c**) two intermolecular H-bonds. Molecular structures of the considered bases.

Neither **3** nor **4** was soluble in weakly polar, aprotic solvents. However, their solubility can be increased in the presence of a dissolved base. Possible scenarios of phthalic acid interaction with bases in solution are shown in Figure 7. If the composition of such acid:base complex is 1:1, one carboxyl group of the acid interacts with the base while the other carboxyl group can either form an intramolecular H-bond with the former one (scenario **a**) or remain free (scenario **b**). If the base is in excess, 1:2 acid:base complex can be formed with two near-equal intermolecular H-bonds (scenario **c)**. We did not consider complexes of acid dimers because such complexes are not likely in the presence of the base because the solubility of the acid alone is very low. What are the individual ^1^H chemical shifts of carboxyl protons in these complexes when the base is very strong? In a polar solvent the ^1^H chemical shifts of the carboxyl proton in a 1:1 complex of 2-nitrobenzoic acid with 2,4,6-trimethylpyridine is equal to 16.8 ppm [93]. The length of this H-bond can be elongated due to the steric effects [94,95]. The use of a stronger base can cause both a contraction and a lengthening of the H-bond. The result depends on the position of the bonding proton with respect to the H-bond center. However, the reduction of solvent polarity causes the opposite effect [66,67,68]. We concluded that the ^1^H chemical shift of the proton in the intermolecular H-bonds in scenarios **a**, **b**, and **c** should have been between 19 and 17 ppm. The ^1^H chemical shift of the proton in the intramolecular H-bond in scenario **a** was the hardest to estimate. The geometry of this H-bond was forced to adapt to the rigid molecular structure. Most likely, the ^1^H chemical shift of this proton should have been smaller than 15 ppm [33]. The ^1^H chemical shift of the proton of the free carboxyl group in scenario **b** depended on interaction with CDCl_3_. At high concentration of 2,6-bis(trifluoromethyl)benzoic acid in dry CDCl_3_, its mobile proton resonates at 10 ppm. At high concentration of 2,6-bis(trifluoromethyl)benzoic acid in toluene its mobile proton resonates at 8.8 ppm at 300 K and at 7.4 ppm at 354 K. In both solvents the chemical shift depends on the monomer–dimer equilibrium of the acid. We believed that 6 ppm was a safe upper limit for the ^1^H chemical shift of the proton of the free carboxyl group in scenario **b**. Summarizing the above, the mean ^1^H chemical shifts of the carboxyl protons in scenario **a**, **c**, and **b** were expected to be about 16 ppm, 18 ppm, and below 12 ppm, respectively.

**Figure 8:**
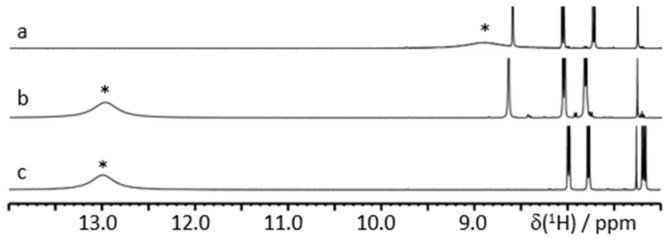
Characteristic ^1^H NMR spectra of **3** and **4** in CDCl_3_ at 300 K in the presence of Et_3_N. The signals of OH-protons are marked by asterisks. The mole fractions are (**a**) water:**4**:Et_3_N = 1:1.2:35, (**b**) water:**4**:Et_3_N = 1:8.7:35, and (**c**) water:**3**:Et_3_N = 1:8.7:35.

Figure 8 shows characteristic ^1^H NMR spectra of **3** and **4** in CDCl_3_ in the presence of a large excess of triethylamine (Et_3_N). The limiting mean values of the ^1^H chemical shift of carboxyl protons measured using a set of spectra collected with a gradual increase in the mole fraction of **3** or **4** were equal to 14.1 ppm for both acids (Tables S1 and S2). Therefore, the most likely structure of a complex of Et_3_N with phthalic acids in nonpolar solvents corresponded to scenario **a**. This result is pretty intuitive while the bulkiness of Et_3_N significantly increased the entropic cost of the structure shown in scenario **c**.

**Figure 9:**
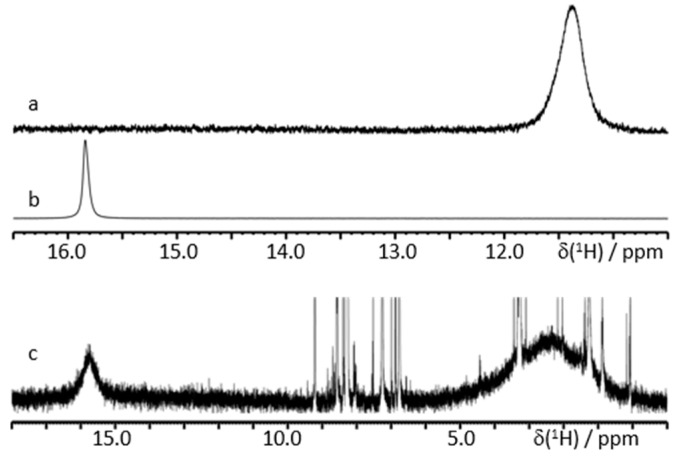
Characteristic ^1^H NMR spectra of **4** in CDCl_3_ at 300 K in the presence of DMAP. The mole fractions are (**a**) water:**4**:DMAP = 1:1.0:260, (**b**) water:**4**:DMAP = 1:3.9:260, and (**c**) water:**4**:DMAP = 1:0.24:0.30.

Figure 9a,b shows characteristic ^1^H NMR spectra of **4** in CDCl_3_ in the presence of a large excess of *N*,*N*-dimethylpyridin-4-amine (DMAP). The limiting mean value of the ^1^H chemical shift of carboxyl protons measured using a set of spectra collected with a gradual increase in the mole fraction of **4** was about 18.5 ppm (Table S3). Therefore, the most likely structure of the complex of DMAP with **4** in CDCl_3_ corresponded to scenario **c**. When the mole fractions of DMAP were only slightly larger than that of **4,** while the total concentration was very low, ^1^H NMR spectra exhibited two separate peaks of different mobile protons (Figure 9c). We attributed the peak at 15.7 ppm to a complex of DMAP with **4** and the peak at 2.4 ppm to water interacting with residual DMAP. The former peak obviously corresponded to the structure in scenario **a**. The mean ^1^H chemical shift in this complex was larger than for Et_3_N. However, this difference does not mean, obviously, that the intermolecular H-bond in DMAP:**4** was stronger than in Et_3_N:**4**. Recall that the ^1^H chemical shift of the strongest known H-bond in [FHF]^−^ is 16.6 ppm [95] while one of the largest ^1^H chemical shifts, of 21.7 ppm, has been measured for a moderately strong H-bond in the proton-bound homodimers of pyridine [33]. More about this issue can be found elsewhere [96]. In contrast, the value of 15.7 ppm in DMAP:**4** can be compared to the value of 18.5 ppm in DMAP:**4**:DMAP. The latter complex has two intermolecular H-bonds while the former has one inter- and one intramolecular H-bond. Therefore, if the effects of mutual influences of the adjacent hydrogen bonds on their geometries in each of these complexes were small, the individual ^1^H chemical shift of the intramolecular H-bond in DMAP:**4** was about 13 ppm.

In contrast to the solution with Et_3_N, the presence of DMAP did not increase the solubility of **3** in CDCl_3_. Presumably, **3** did not interact with DMAP by scenario **c** due to a high entropic cost caused by the position of the nitro group. Why did it not interact with DMAP by scenario **a**? We cannot answer this question with certainty.

### 2.4. H-Bonding Vibrational Modes in Carboxyl Dimers

Stretching vibrations of H-bonds have a high diagnostic value for determination of the nature and strength of these bonds [97,98]. Previously, the spectral manifestations of dimerization and isotopic effects on spectroscopic observables were studied for different molecular systems [99,100,101,102,103,104,105,106,107,108] including carboxylic acid dimers [109,110]. Upon carboxylic acid dimerization, the structure of the OH stretching band in IR spectra changes most prominently: The narrow band of monomers changes to a broad, intensive, and complex substructured band of dimers shifted to lower wavenumbers.

**Figure 10:**
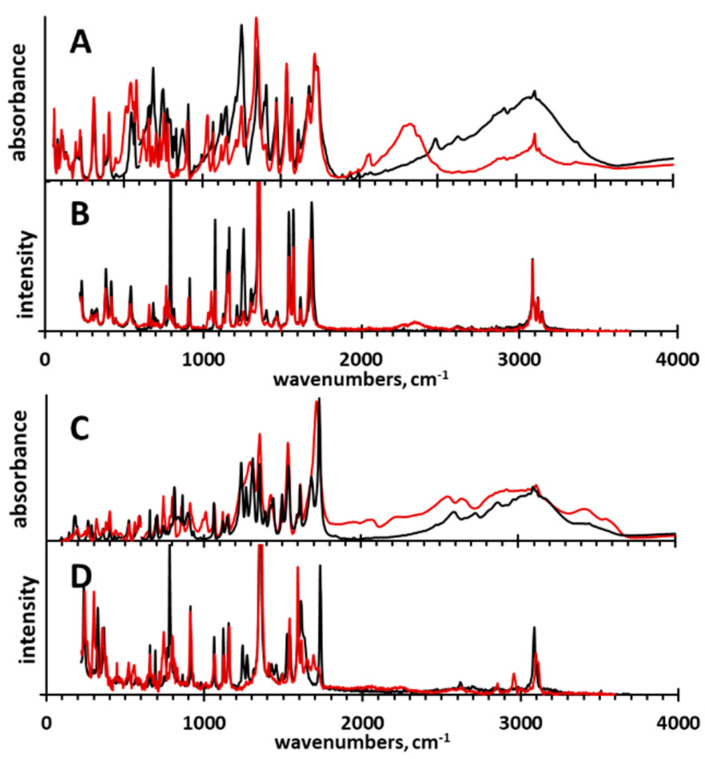
Normalized experimental IR and Raman spectra of **3** (**A** and **B**) and **4** (**C** and **D**) (black spectra) and their deuterated (OD) derivatives (red spectra).

**Figure 11:**
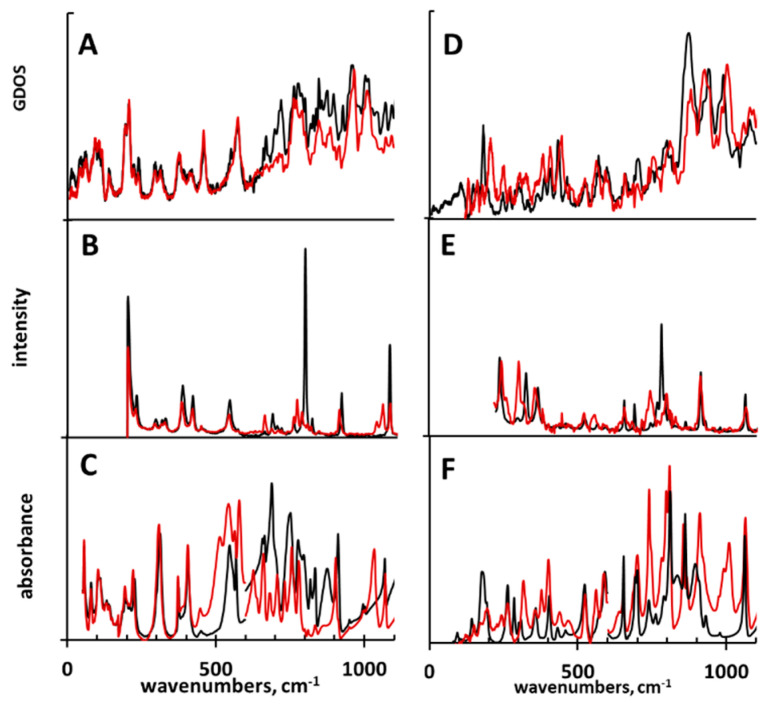
Normalized IINS (**A**, **D**), Raman (**B**, **E**), and IR (**C**, **F**) spectra of compounds **3** (**A**—**C**, black spectra) and **4** (**D**–**F**, black spectra) and their deuterated derivatives (red spectra).

For a comprehensive spectroscopic investigation of **3** and **4**, we accomplished a study based on IR, Raman, and IINS measurements, as well as DFT, CPMD, and Potential Energy Distribution (PED) calculations. The IR, Raman, and IINS spectra of non-deuterated and deuterated (OH → OD replacement) **3** and **4** are shown in Figure 10 and Figure 11. The experimental spectra were interpreted using the calculated vibrational (DFT) and power spectra (CPMD), and the results of the PED analysis (Tables S4 and S5). More about this issue can be found elsewhere [111,112,113].

According to the crystallographic data [37], the molecules of **3** form H-bonded oligomeric chains of dimers. These H-bonds were almost of the same length (Table 1) and, consequently, were equally strong. Therefore, the stretching vibrations of the OH group (ν(OH)) were within the same spectral range and overlapped in the experimental IR spectra (Figure 10). The shapes of the ν(OH) and ν(OD) bands were very alike to those of carboxylic acid dimer studied experimentally and theoretically by Flakus et al. [110]. For **3**, the deuteration caused a shift of the ν(OD) band to lower wavenumbers according to the well-established rulewith the isotopic spectroscopic ratio ISR = δ_OH_/δ_OD_ = 1.28 [114]. In contrast, for **4** the band ν(OD) expanded strongly; this revealed a complex character of the underlying changes. Deformational (δ(OH)/δ(OD) and γ(OH)/γ(OD)) bands are informative because in **3** and **4** they differed from those observed for intramolecular H-bonds in *ortho*-hydroxy aryl Schiff bases [115,116,117,118] and *ortho*-acetophenones [119]. The δ(OH) was a doublet at 1409/1395 cm^−1^ in **3** and a band at 1383 cm^−1^ in **4**. Upon deuteration, these bands disappeared to emerge at 1033/1029 cm^−1^ in **3** and at 1014/995 cm^−1^ in **4**. Thus, the ISR is in the range of 1.36–1.35 for both compounds (Tables S4 and S5). This characteristic behavior of the ISR deviated from that of *ortho*-hydroxy aryl Schiff bases [115] and *ortho*-hydroxy acetophenones [119]. When it comes to the bands assigned to the deformational γ(OH) vibrations, a few bands shifted to the low wavenumbers’ region after the deuteration: 876, 835, 819, 796, 752, and 691 cm^−1^ for **3** and 862, 839, and 763 cm^−1^ for **4** in IR/Raman spectra (Tables S4 and S5). The assignments of these bands to the deformational vibrations of the bridging protons was unequivocal because the intensity of the two series of the bands at 895, 875, 845, 826 cm^−1^ and 778, 715, 668 cm^−1^ for **3** and 874 and 704 cm^−1^ for **4** was greatly decreased in the IINS spectra after the deuteration (Figure 11 and Table S4). This phenomenon has been studied in the past [120,121,122,123,124,125]. As for the emergence of the two series of bands assigned to the deformational vibrations, it can be explained by the presence of the dimers and the oligomers in the solid state (see above). The attribution of two deformational bands to dimers and monomers was suggested by Miyazawa and Pitzer [126] for formic acid in the gas phase and solid nitrogen matrixes. Thus, the two series of the (γ(OH) bands at 876 cm^−1^ (γ(OD) = 627 cm^−1^), 835 cm^−1^ (627 cm^−1^), 819 cm^−1^ (627 cm^−1^), and 796 cm^−1^ (580 cm^−1^) and at 752 cm^−1^ (545 cm^−1^), 690 cm^−1^ (514 cm^−1^), and 874, 704 cm^−1^ are assigned to the deformational vibrations of the carboxyl groups of the dimers and the monomers of **3** and **4**, respectively.

The assignment of γ(OH) can be supported by the previously published d(OO) = f(γ(OH)) correlation [127,128] and the crystallographic data for **3** [37]. The lengths of the H-bridges in the range of 2.65–2.70 Å (*d*(OO) is calculated by means of the *d*(OO) = 3.01–4.4 × 10^−4^ γ(OH) correlation, where *d*(OO) is in Å and γ(OH) is in cm^−1^) matched very well with the experimentally measured ones (*d*(OO) = 2.698 Å and 2.681 Å [37]). Moreover, the experimentally obtained wavenumber values for the γ(OH) bands can be applied to compare the strength of the H-bonds in **3** and **4**. The obtained results show that the H-bonds in **3** were a bit weaker than in **4** (*d*(OO) is in the range of 2.65–2.70 Å for **3** and 2.63–2.67 Å for **4**), though the difference in the strength of the hydrogen bonding was not large.

In terms of the H-bond vibrations, the IINS spectroscopy allows one to unequivocally interpret bands (ν_σ_) due to the almost complete disappearance of these bands upon deuteration [115,121]. Based on this phenomenon, two low-intensity bands at 555 and 390 cm^−1^ were assigned to vibrations ν_σ_^asym^ and ν_σ_^sym^ of the H-bonds, respectively. Importantly, these bands overlapped with the bands of other vibrations (insensitive to deuteration) both in IINS and IR spectra. However, the changes of the IINS spectra were much clearer than ones of the IR spectra.

**Figure 12:**
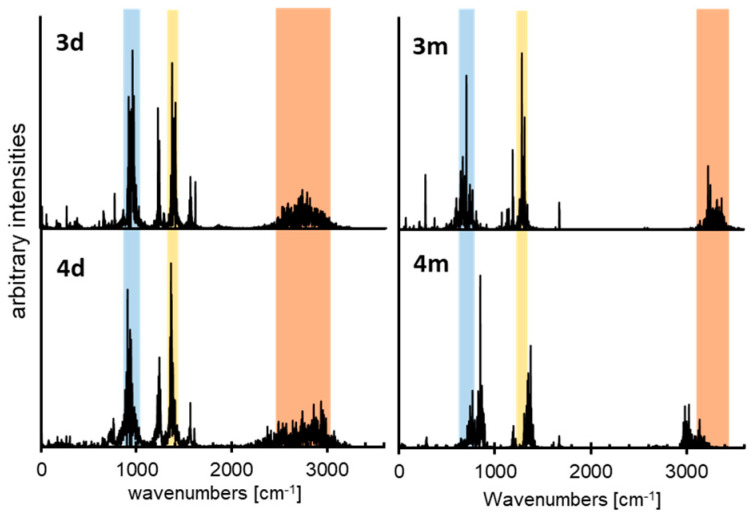
Calculated power spectra of atomic velocity–results of the CPMD runs for the monomers of **3** and **4** (**3m** and **4m**) as well as for the dimers of **3** and **4** (**3d** and **4d**). The CPMD power spectra are presented only for the bridged protons vibrational modes. The stretching vibration area is shown in red. The bending vibration areas are shown in blue and yellow.

The relative strengths of the H-bonds in dimers and monomers of **3** and **4** can be evaluated using atomic velocity power spectra obtained from the CPMD trajectories. The vibrational spectra related to the atomic motion intensity (arbitrary intensities) are presented in Figure 12. The bands of hydroxyl groups are relatively broad (red bars in Figure 12): 3100–3500 cm^−1^ and 2920–3250 cm^−1^ for the monomers of **3** and **4** and 2400–3100 cm^−1^ and 2250–3200 cm^−1^ for the dimers of **3** and **4**. The bands of the dimers are strongly red-shifted as compared to those of the monomers. This shift indicates that the intermolecular H-bonds in the dimers were much stronger than the intramolecular ones in the monomers.

The stretching and bending vibration areas of the dimers of **3** and **4** did overlap (Figure 12, **3d** and **4d**). In contrast, the stretching and bending vibration bands of the monomer of **4** were blue- and red-shifted, respectively, as compared to those of 3 (Figure 12, **3m** and **4m**). Therefore, the strengths of H-bonds in the dimers of **3** and **4** were similar. In contrast, the intramolecular H-bond in the monomer of **3** was weaker than that in the monomer of **4**. The reason for that is that the structure of the monomer of **3** was more bent. This conclusion is consistent with the above interpretation of the experimental data and demonstrates that experimental spectroscopic studies and CPMD simulations greatly enhance each other’s results.

## 3. Materials and Methods

### 3.1. Compounds and Deuteration

The studied compounds and solvents were purchased from Sigma-Aldrich company and used without further purification. The deuterated sample was prepared by dissolving the product in deuterated methanol (CH_3_OD). The solution was then heated to 60 °C and refluxed during 30 min. After that, the methanol was removed by evaporation under reduced pressure. This procedure was repeated three times.

### 3.2. Infrared and Raman Measurements

The far and middle infrared (FIR, MIR) absorption measurements were performed using *a* Bruker Vertex 70v vacuum Fourier Transform spectrometer. The transmission spectra were collected with a resolution of 2 cm^−1^ and with 64 and 32 scans per each spectrum for FIR and MIR, respectively. The FT-FIR spectra (500–50 cm^−1^) were collected for the samples suspended in Apiezon N grease and placed on a polyethylene (PE) disc. The FT-MIR spectra were collected for the samples in a KBr pellet. The Raman spectra of the analyzed samples were obtained using FT-Nicolet Magma 860 spectrophotometer The In:Ga:Ar laser line at 1064 nm was employed for the Raman excitation measurements. The spectra were recorded at the room temperature in the range of 200–3800 cm^−1^ with the spectral resolution of 4 cm^−1^ and with the same number of scans (512/measurement).

### 3.3. Incoherent Inelastic Neutron Scattering (IINS) Measurements

Neutron scattering data were collected at the pulsed IBR-2 reactor at the Joint Institute of Nuclear Research (Dubna) using the time-of-flight inverted geometry spectrometer NERA at 10 K temperature. The spectra were converted from neutron per channel to the scattering function per energy transfer. At the energy transfer between 5 and 1200 cm^−1^, the relative IINS resolution was estimated to be ca. 3%. The *S*(***Q***, *ω*) function (scattering law) can be expressed in the form of isotropic harmonic oscillator [129]:

(1) $$S\left(Q,n\omega \right)=\frac{\left(Q^{2}\cdot U^{2}\right)}{n!}\cdot exp\left(\left(Q^{2}\cdot U^{2}\right)\right)$$

where ***Q*** is the momentum transfer and *U*^2^ is the mean square displacement defined as

(2) $$U^{2}=\frac{\hbar }{2m\omega }=\frac{16.795}{\mu \nu }$$

where *µ* is the mass oscillator in amu, ν is the oscillator energy in cm^−1^, *U*^2^ is expressed in Å^2^, and *n* is the number of excited states.

### 3.4. NMR Measurements

The ^1^H spectra were recorded at room temperature on a Bruker Avance III 500 MHz spectrometer. CDCl_3_ was purchased from Sigma-Aldrich and used without further purification. The spectra were measured using the solvent peak as an internal reference, and the chemical shifts were converted to the conventional TMS scale. The number of scans varied between 128 and 256.

### 3.5. Car–Parrinello Molecular Dynamics’ Simulations

A dynamical nature of the investigated molecules **3** and **4**, with the emphasis on their hydrogen bridges, was studied using Car–Parrinello molecular dynamics (CPMD) [130]. The models of monomers and dimers for the CPMD simulations were constructed on the basis of static DFT gas phase results. The molecular structures were placed in cubic boxes with *a* = 15 Å for the monomeric forms and *a* = 22 Å (for compound **3**) and *a* = 25 Å (for compound **4**) for dimeric forms. The first-principle molecular dynamics (FPMD) calculations were performed in the gas phase with the empirical van der Waals correction by Grimme (all DFT-D2) [131]. The Perdew–Burke–Ernzerhof (PBE) exchange-correlation DFT functional [132] was applied. The core electrons of the studied monomers and dimers were replaced by norm-conserving pseudopotentials of Troullier–Martins type [133]. The Kohn–Sham orbitals were expanded using the plane-wave basis set with the maximum kinetic energy cutoff of 90 Ry. The Hockney’s scheme [134] was used to remove interactions with periodic images and simulate isolated molecule conditions. The orbital coefficients were propagated using the default value of the fictitious orbital mass, 400 a.u., and the nuclear motion timestep was set to 2 a.u. The CPMD simulations were divided into two steps: The equilibration and the production runs. During the equilibration, the ionic temperature was set to 297 K and controlled by Nosé–Hoover thermostat chains with default settings, with each degree of freedom coupled to a separate thermostat (“massive” thermostatting) [135,136]. The Nosé–Hoover thermostat chain was set to 3200 cm^−1^ frequency. The equilibration runs of the CPMD lasted for 50,000 steps for the monomers and dimers. The data collection lasted for 500,000 steps (24 ps) using the NVE microcanonical ensemble (the thermostat chains were detached during the simulations). The obtained trajectories served as a basis for the distance evolution analysis of the bridged proton and the functional groups’ dynamic as well as to determine the vibrational features of the investigated compounds from the power spectra of atomic velocity.

The CPMD simulations were carried out using the CPMD 3.17.1 program [137]. The data analysis was performed using locally written utilities and the VMD 1.9.3. program [138]. The graphical presentation of the obtained results was prepared with the Gnuplot graphics package [139], and with the VMD 1.9.3. program [138].

### 3.6. DFT Calculations

This part of the calculations was performed with the Gaussian 09 suite of programs [140] using the density functional theory (DFT) with the three-parameter functional proposed by Becke with the correlation energy according to the Lee–Yang–Parr formula, denoted as B3LYP [141,142]. The triple-zeta split-valence basis set, denoted as 6-311+G(d,p) [143,144,145] according to the Pople’s notation, was applied. The use of diffuse functions is a proper approach for studies of hydrogen bonding [146]. Initially, the geometry optimization was carried out and followed by harmonic frequencies’ calculations, confirming that the obtained structures correspond to the minima on the potential energy surface (PES). Next, the one-dimensional reaction path of the bridged proton transfer from donor to the acceptor atom within the intramolecular hydrogen bond was studied. The applied approach was based on stepwise elongation of the O-H distance (with 0.05 or 0.1 Å increments) with full optimization of the remaining structural parameters. The calculations were carried out in the gas phase and with the solvent reaction field using acetonitrile as a solvent. The Polarizable Continuum Model (PCM) method [147] was used to reproduce the solvent influence on the studied molecules. All the performed calculations were conducted for the electronic ground state and without any extra charges on the molecules and dimers. The obtained results were visualized using the MOLDEN software [148].

### 3.7. PED Analysis

The potential energy distribution (PED) of the normal modes was calculated in terms of natural internal coordinates [149] using the Gar2ped program [150].

## 4. Conclusions

The result of the interplay between competing noncovalent interactions in the condensed phase may appear to be quite unexpected. The conformation of carboxyl groups is assumed to be dominantly *cis* due to so-called *Z*-effect [59]. However, the conformation can be changed in H-bonded associates [56,57,58,59]. We herein reported a comprehensive computational and experimental study of this phenomenon using 3-nitrophthalic (**3**) and 4-nitrophthalic acids (**4**) as model systems. It was observed that an intermolecular H-bond interaction between the adjacent carboxyl groups of these molecules became favorable only when one of the groups was involved in a strong intermolecular H-bond. However, even in this case, the spatial distance between the carboxyl groups needed to be increased. If the latter was not possible, for example due to steric hindrances, as in **3**, the intramolecular interaction was energetically unfavorable. As a result, the intramolecular steric hindrances critically affected the solubility, the crystal packing, and the intramolecular proton exchange of phthalic acids.

The structural and energetic parameters of intra- and intermolecular interactions in the monomers, dimers, and aggregates of **3** and **4** were estimated for the gas, liquid, and solid phases.

## Figures and Tables

**Table 1:** Metric parameters (in Å) for the donor-acceptor (OO) and donor-proton (OH) contacts in the monomers and dimers of **3** and **4**. The CPMD results are given as: Average ± standard deviation.

Compound	Method	Bridge 1		Bridge 2			
		d(OH)	d(OO)	d(OH)	d(OO)	OHO[°]	COH^…^O[°]
**3**, monomer	CPMD	0.993 ± 0.022	2.728 ± 0.151	-	-	-	-
**3**, dimer	-	1.027 ± 0.032	2.634 ± 0.095	1.028 ± 0.034	2.633 ± 0.091	-	-
**4**, monomer	-	1.005 ± 0.022	2.587 ± 0.088	-	-	-	-
**4**, dimer	-	1.028 ± 0.036	2.650 ± 0.122	1.028 ± 0.037	2.653 ± 0.115	-	-
**3**, monomer	DFT	0.978	2.670	-	-	150.2	64.4
**3**, dimer	-	0.999	2.679	1.001	2.660	178.9	0.2
**4**, monomer	-	0.985	2.583	-	-	160.0	50.1
**4**, dimer	-	1.000	2.669	0.999	2.679	178.6	0.7
**3**, dimer	X-ray [38]	0.84	2.698	0.84	2.698	155.5	-
**3**, oligomer	-	0.84	2.681	-	-	-	-

## Supplementary Materials

The following are available online, Figure S1: The dimeric forms of compounds **3** and **4** and relative energy values obtained at B3LYP/6-311+G(d,p) level of theory, Figure S2: Structures and atoms numbering of studied compounds **3** and **4**, Figure S3: Calculated potential energy curves for the gradual nitro group rotation of conformers **3(I)** and **4(II)**, Figure S4: Calculated (B3LYP/6-311+G(d,p), PCM approach for acetonitrile (**a**) and gas phase (**b**)) potential energy functions by the gradual displacement of one proton for compounds **3** and **4** whereas the remaining parameters were optimized: in the intramolecular hydrogen bond of monomers, in the intermolecular hydrogen bond of dimers and in the intermolecular hydrogen bond of dimers for fixed adjacent bridged proton; Figure S5: Time evolution of the metric parameters of two symmetric hydrogen bridges. The CPMD gas phase simulations of the dimers of **3** and **4**. Donor-proton distance, proton-acceptor distance, donor-acceptor distance; Table S1: ^1^H NMR data for compound **3** in CDCl3 in the presence of *N*,*N*-diethylethanamine (Et3N), Table S2: ^1^H NMR data for compound **4** in CDCl3 in the presence of *N*,*N*-diethylethanamine (Et3N), Table S3: ^1^H NMR data for compound **4** in CDCl3 in the presence of *N*,*N*-dimethylpyridin-4-amine (DMAP), Table S4: Experimental IR, Raman, IINS and calculated DFT (B3LYP/6-311+G(d,p)) spectral data of compound **3** and its mono deuterated (OH→OD) derivative, Table S5: Experimental IR, Raman, IINS and calculated DFT (B3LYP/6-311+G(d,p)) spectral data of compound **4** and its mono deuterated (OH→OD) derivative.
