# Supplementary material for: Inter- vs. Intramolecular Hydrogen Bond Patterns and Proton Dynamics in Nitrophthalic Acid Associates
Source: Molecules. 2020 Oct 14;25(20):4720. doi: 10.3390/molecules25204720 (PMC7587347; doi:10.3390/molecules25204720)
Supplement: Supplementary file 1 [file molecules-25-04720-s001.pdf]

molecules

MDPI

Supporting Information for Publication

Inter- vs. Intramolecular Hydrogen Bond Patterns and Proton Dynamics in Nitrophthalic Acid Associates

Kinga Jóźwiak [1], Aneta Jezierska [1], Jarosław J. Panek [1], Eugene A. Goremychkin [2], Peter M. Tolstoy [3], Ilya G. Shenderovich [4,*] and Aleksander Filarowski [1,*]

[1] Faculty of Chemistry, University of Wrocław 14 F. Joliot-Curie str., 50-383 Wrocław, Poland; kin.joz@o2.pl, aneta.jezierska@chem.uni.wroc.pl, jaroslaw.panek@chem.uni.wroc.pl, aleksander.filarowski@chem.uni.wroc.pl
[2] Frank Laboratory of Neutron Physics, Joint Institute for Nuclear Research 6 F. Joliot-Curie str., 141980 Dubna, Russia; goremychkin@jinr.ru
[3] Institute of Chemistry, St. Petersburg State University, Universitetskij pr. 26, 198504 St. Petersburg, Russia; peter.tolstoy@spbu.ru
[4] Institute of Organic Chemistry, University of Regensburg, Universitaetstrasse 31, 93053 Regensburg, Germany; Ilya.Shenderovich@chemie.uni-regensburg.de

* Correspondence: aleksander.filarowski@chem.uni.wroc.pl; Tel.: +48-71-375-7229 (A.F.)
Ilya.Shenderovich@chemie.uni-regensburg.de (I.G.S.)

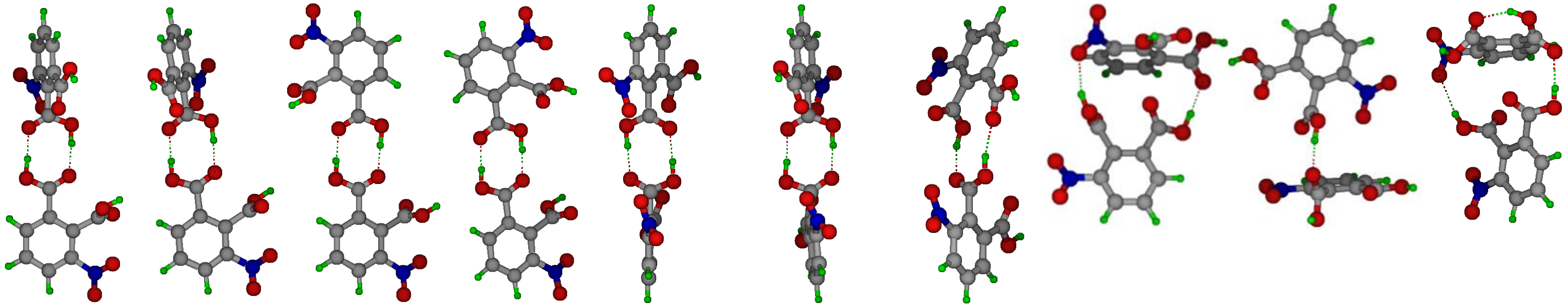

**D3(I)**	**D3(II)**	**D3(III)**	**D3(IV)**	**D3(V)**	**D3(VI)**	**D3(VII)**	**D3(VIII)**	**D3(IX)**	**D3(X)**
ΔE(**D3**(**I**))	ΔE(**D3**(**II**))	ΔE(**D3**(**III**))	ΔE(**D3**(**IV**))	ΔE(**D3**(**V**))	ΔE(**D3**(**VI**))	ΔE(**D3**(**VII**))	ΔE(**D3**(**VIII**))	ΔE(**D3**(**IX**))	ΔE(**D3**(**X**))
0.00	0.06	0.10	0.63	1.16	1.55	6.03	8.32	6.42	8.54

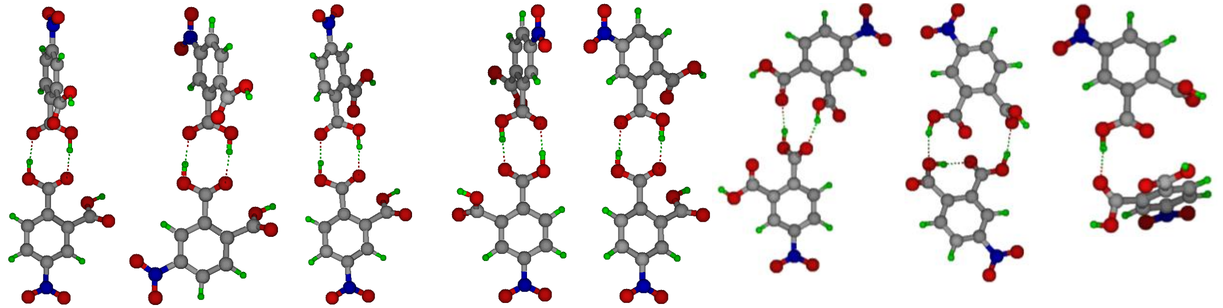

**D4(I)**	**D4(II)**	**D4(III)**	**D4(IV)**	**D4(V)**	**D4(VI)**	**D4(VII)**	**D4(VIII)**
ΔE(**D4**(**I**))	ΔE(**D4**(**II**))	ΔE(**D4**(**III**))	ΔE(**D4**(**IV**))	ΔE(**D4**(**V**))	ΔE(**D4**(**VI**))	ΔE(**D4**(**VII**))	ΔE(**D4**(**VIII**))
0.00	0.03	0.05	0.36	0.36	3,96	4,84	6.84

Figure S1. The dimeric forms of compounds **3** and **4** and relative energy values (in kcal/mol) obtained at B3LYP/6-311+G(d,p) level of theory.

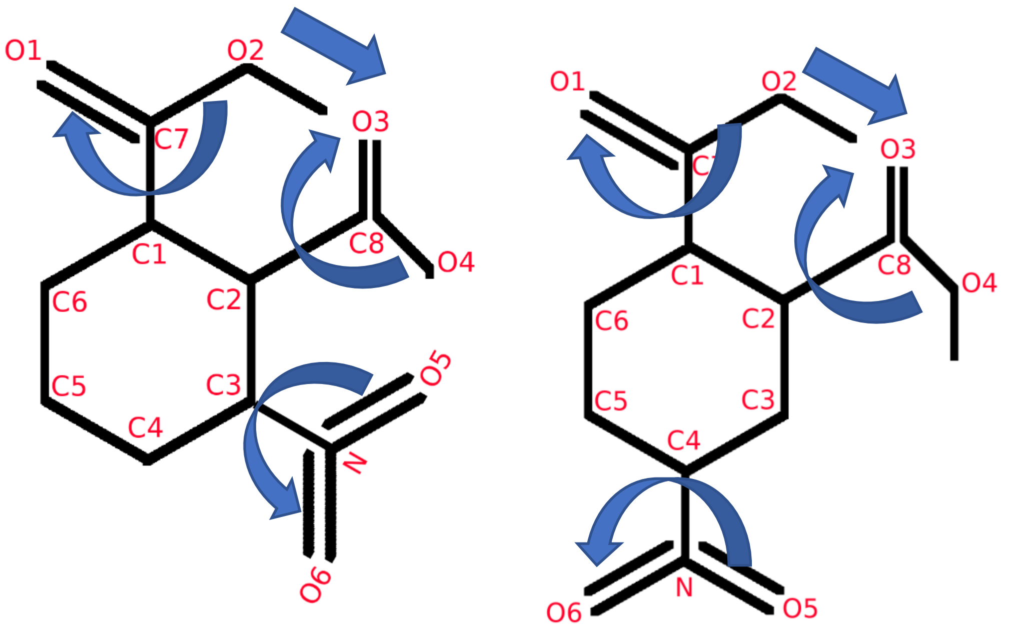

Figure S2. Structures and atoms numbering of studied compounds **3** and **4**.

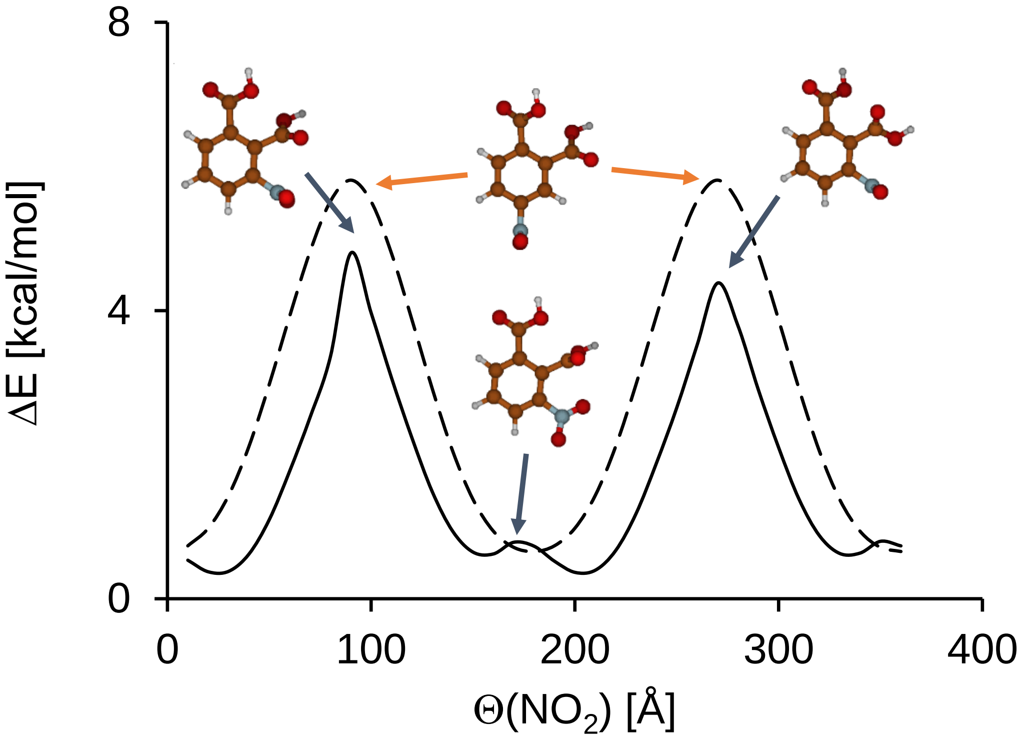

Figure S3. Calculated potential energy curves for the gradual nitro group rotation of conformers **3(I)** (solid line) and **4(II)** (dashed line).

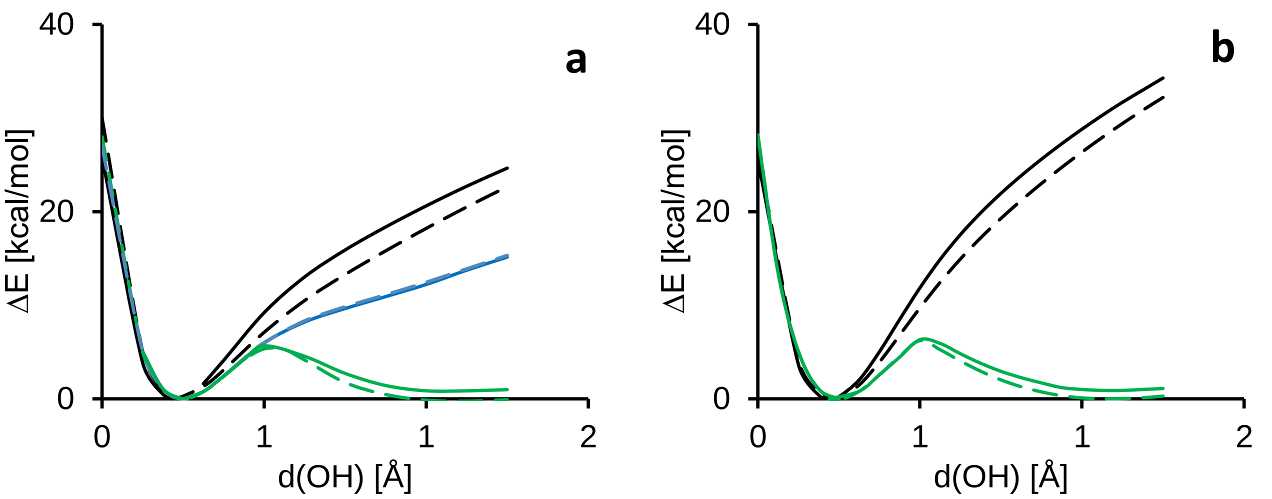

Figure S4. Calculated (B3LYP/6-311+G(d,p), PCM approach for acetonitrile (**a**) and gas phase (**b**)) potential energy functions by the gradual displacement of one proton for compounds **3** (solid lines) and **4** (dashed lines) whereas the remaining parameters were optimized: in the intramolecular hydrogen bond of monomers (black lines), in the intermolecular hydrogen bond of dimers (green lines) and in the intermolecular hydrogen bond of dimers for fixed adjacent bridged proton (blue lines).

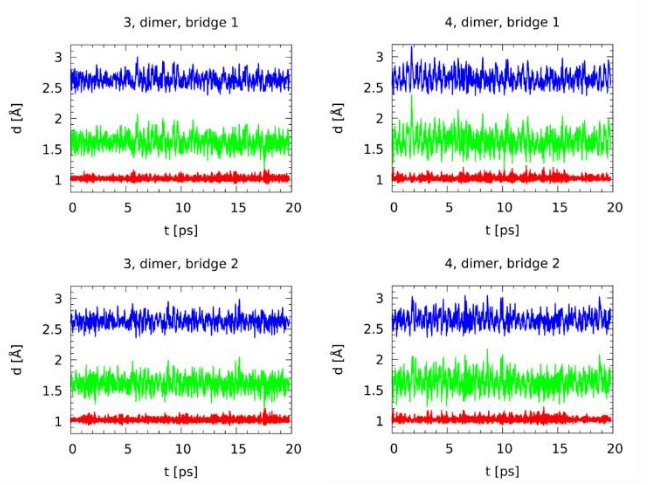

Figure S5. Time evolution of the metric parameters of two symmetric hydrogen bridges. The CPMD gas phase simulations of the dimers of **3** and **4**. Red: donor-proton distance, green: proton-acceptor distance, blue: donor-acceptor distance.

Table S1. ^{1}H NMR data for compound **3** in $CDCl_3$ in the presence of N,N-diethylethanamine (Et_3N).

Mean chemical shift of mobile protons/ ppm	Et_3N mole fraction	Water mole fraction	compound **3** mole fraction	Estimated mean chemical shift of the mobile protons of compound **3**/ ppm
3.3	35	1	0	-
13.0	35	1	8.7	14.1
13.4	35	1	15.1	14.1
13.4	70	1	15.1	14.1

Table S2. ^{1}H NMR data for compound **4** in $CDCl_3$ in the presence of N,N-diethylethanamine (Et_3N).

Mean chemical shift of mobile protons/ ppm	Et_3N mole fraction	Water mole fraction	compound **4** mole fraction	Estimated mean chemical shift of the mobile protons of compound **4**/ ppm
3.3	35	1	0	-
8.9	35	1	1.2	13.6
11.9	35	1	4.0	14.1
13.0	35	1	8.7	14.1
13.3	35	1	13.2	14.1
13.1	70	1	13.2	13.8

Table S3. ^{1}H NMR data for compound **4** in $CDCl_3$ in the presence of N,N-dimethylpyridin-4-amine (DMAP).

Mean chemical shift of mobile protons/ ppm	DMAP mole fraction	Water mole fraction	compound **4** mole fraction	Estimated mean chemical shift of the mobile protons of compound **4**/ ppm
4.58	260	1	0	-
11.38	260	1	1.04	17.9
13.31	260	1	1.75	18.3
14.63	260	1	2.44	18.7
15.31	260	1	3.23	18.6
15.84	260	1	3.85	18.8

molecules

MDPI

Table S4. Experimental IR, Raman, IINS and calculated DFT (B3LYP/6-311+G(d,p)) spectral data of compound **3** and its mono deuterated (OH → OD) derivative.

№	IR(OH), cm^{-1}	IR(OD), cm^{-1}	R(OH) cm^{-1}	R(OD) cm^{-1}	IINS (OH), cm^{-1}	IINS (OD) cm^{-1}	DFT [cm^{-1}]	Int. [km/mol]	Potential energy distribution (%)
1					20	~14			
2					44	42	32.15	0.5	τ(C8OO) 47; τ(C7OO) 25; τ(NO_2) 19
3	55,4	55,4			60	62	53.08	0.29	τ(NO_2) 40; τ(CCC) 25; τ(C7OO) 22
4	80,0	80,0			94	92,5	78.67	1.6	γ(C2-C8) 31; ϱ(C8OO) 26; τ(CCC) 10
5	104,1	104,1			106	107			
6	109,9	109,9			114	115			
7	132,6	132,6							
8	140,3	140,3			140	141	144.92	1.63	τ(C8OO) 76
9							164.17	1.36	γ(C1-C7 + C2-C8 – C3-N) 47; ϱ(C1-C7 + C3-N + C8OO) 28
10							168.05	1.74	γ(C1-C7 + C2-C8 – C3-N) 43; ϱ(C1-C7 + C3-N + C8OO) 31
11							175.37	2.68	ϱ(C2-C8 + C3-N) 69; ω(C8OO) 9
12	187,6	187,6 w					180.51	0.95	τ(CCC) 39; γ(C1-C7 + C3-N + C4-H) 32
13	195,3	193,3			194	195			
14	211,7 w	208,8 w			206	206			
15					224	221			
16		221,7		227					
17	223,2		231		240	239 w			
18							280.46	1.41	ϱ(C1-C7 + C2-C8 – C3-N) 50; ϱ(NO_2 – C7OO) 28; ω(C8OO) 10
19			294	292	294	295	289.6	2.09	δ(CCC) 37; ν_{asym}(C2-C8 – C3-N – C1-C7) 31
20	311,5	309,1	316	314	314	312			
21			327	325			350.37	1.06	τ(CCC) 40; γ(C1-C7 – C3-N) 23; ϱ(C8OO) 15

22	374,1	373			378	378	379.61	3.01	ϱ(NO_2 + C7OO) 22; τ(CCC) 19; ν(C2-C8) 9
23			385	381					
24		392,0							
25	408,9				390	-			
26	408,9	406,9	418	418	412	420	403.59	2.35	δ(CCC) 40; ν_{asym}(C3-N – C1-C7) 31
27	451,3	453,7	446	446	459	457	441.61	5.02	τ(CCC) 43; γ(C1-C7 + C3-N) 36
28		514,9					509.91	16.25	τ(C8-O4-H) 31; ϱ(C1-C7 + C7OO) 31; ω(C8OO) 10
29	548,2	544,8	542	537	555	-	541.59	38.23	ϱ(NO_2 + C7OO) 33; τ_{sym}(C7OO + C8OO) 17; δ(C8OO) 6
30							570.15	30.91	τ(CCC) 57; τ_{asym}(C7OO – C8OO) 19
31							588.76	33.18	δ_{asym}(C7OO – C8OO) 23; τ(CCC) 20; ϱ(C8OO) 18
32		565,6 w							
33	568,5		570		572	572	599.23	76.68	τ(C-O-H) 46; γ(C1-C7 + C3-N) 12; ϱ(NO_2) 11
34		580,0				-			
35		626,8				-			
36		640 sh				637			
37	657,7			659			640.01	45.75	δ_{asym}(C7OO – C8OO) 55; δ(CCC) 25
39	664,5	661,6			668				
40		683,7	684	681			674.77	68.45	δ_{sym}(C7OO + C8OO) 43; δ(CCC) 23
41	690,5		699						
42	702,1	707,3		704	715				
43			714 w	714			714.01	30.66	τ(CCC) 38; ω(NO_2) 18; γ(C2-C8) 10; ω(C7OO) 9
44		732,2		734 w			746.86	54.85	ω(C8OO) 31; ω(NO_2) 10; ϱ(C2-C8) 9; δ(CCC) 8
45	752,2		756				767.36	15.81	γ(C2-C8 + C3-N + C5-H) 35; τ(CCC) 22; ω(NO_2) 10; ω(C8OO) 8
46		755,1		766	760	766	778.25	14.24	δ(NO_2 + C8OO) 21; δ(CCC) 16; ν(C2-C8) 12
47	778,3	780,2		782			785.93	48.72	γ(C1-C7 – C5-H – C6-H) 39; ω(C7OO) 28; ω(NO_2) 9
48	795,6	800,4 w	793		778	-			
49				800 w	795	791,2			

50	818,8		817	812 w	826	-			
51	835,2	835,2 w		839	845		813.81	0.73	δ(CCC) 32; δ(NO_2) 23; ν(C1-C7) 10
52						845	847.65	2.51	γ(C1-C7 – C3-N + C4-H + C5-H) 40; τ(CCC) 29
53	875,7	-			875				
54						885			
55				906	895				
56	911,3	903,6	913	913sh	926	916 w	913.57	27.25	ν_{asym}(C3-N – C1-C7 – C7-O2) 39; δ(NO_2) 29; δ(CCC) 10
57	948,5	948,5			960	966	969.13	0.5	γ(C-H) 86; τ(CCC) 7
58	995,2	995,2							
59	1010,6 w	1010,6 w			1007	1007	1015.78	1.48	γ(C-H) 80; τ(CCC) 13
60		1029,0 sh							
61		1032,8		1032					
62				1051					
63	1068,5	1071,4	1075	1076			1079.53	71.9	δ(CCC) 33; ν_{asym}(C8-O4 – C7-O2) 20; ν_{sym}(C4-C5 + C5-C6) 20
64	1120,6	1119,6	1127	1129			1126.33	195.42	ν(C7-O2) 32; ν_{asym}(C3-N – C4-C5 – C5-C6) 28
65							1133.3	118.8	ν(C8-O4) 31; ϱ(C-H) 22; ν_{sym}(C4-C5 + C7-O2) 12
66	1154,4	1153,4	1156	1154			1183.76	69.17	ϱ(C5-H) 28; ν(C4-C5) 10; δ(CCC) 9; δ(C7-O2-H) 8
67			1164	1164			1198.76	105.96	δ(C7-O2-H) 26; ϱ(C4-H – C5-H) 25; ν_{asym}(C1-C7 – C7-O2) 21
68	1214,1	1214,1	1214	1214			1210.5	177.78	δ(C8-O4-H) 40; ν_{asym}(C2-C8 – C8-O4 – C3-C4) 31
69	1249,8	1249,9	1248 sh				1236.6	11.03	ϱ(C-H) 43; ν_{asym}(C3-C4 – C1-C6) 18; ϱ(C2-C8) 10
70			1256	1254					
71	1302,9	1302,9 w	1303	1302					
72	1321,2	1321,2 w	1320				1333.31	7	ν_{asym}(C1-C2 – C2-C3 +C3-C4 – C4-C5 + C5-C6 – C1-C6) 86
73		1344,3					1361.76	109.07	δ(C-O-H) 25; ν(C7-O2 + C8-O4) 16; ν(C1-C7) 4
74	1356,9	1354,9	1351	1351			1364.36	51.51	δ(C-O-H) 28; ν(C7-O2 + C8-O4) 20; ν(C1-C7) 12
75	1395,4	1398,3 w					1377.95	228.62	ν_{sym}(ONO) 62; δ(NO_2) 16; ν(C3-N) 13
76	1409,0	1404,1 w	1400	1405 w					

77			1468	1468	1471.58	9.25	ϱ(C-H) 46; ν_{asym}(C2-C3 + C3-C4 – C5-C5 – C1-C6) 35
78	1473,6	1471,6			1491.56	28.94	ϱ(C-H) 35; ν_{asym}(C1-C2 + C2-C3 – C3-C4) 29; ν(C1-C7) 6
79	1540,1	1540,1	1542	1542			
80	1570,0	1570,0	1571	1571	1596.99	174.93	ν_{asym}(ONO) 65; ν(C5-C6) 10
81	1612,4	1612,4	1616	1615	1608.15	82.23	ν_{sym}(C1-C2 + C4-C5) 43; ϱ(C-H) 13
82	1665,5	1656,8 w					
83	1682,8	1675,1	1687	1676	1649.83	47.82	ν_{asym}(C2-C3 – C3-C4 + C5-C6 – C1-C6) 52; ν_{asym}(ONO) 16
84	1719,5	1719,5			1803.36	270.39	ν(C7=O1) 79
85	1739,9	1739,9			1818.91	387.01	ν(C8=O3) 84
86		2300		2341			ν(O-D)
87				2610			ν(O-D)
88			2617		3753.14	109.04	ν(O4-H) 100
89	2950		2700		3767.78	125.98	ν(O2-H) 100
90			3084	3084			
91			3095	3094	3193.53	3.43	ν_{asym}(C-H) 95
92	3114		3118	3119	3219.99	3.74	ν_{asym}(C-H) 99
93		3150,6	3144	3145	3222.77	0.96	ν_{sym}(C-H) 100

ν - stretching vibration; δ - scissoring vibration; ρ - rocking; ω - wagging; γ – out-of-plane; τ - twisting and torsional vibration; sh - shoulder and w – weak (small intensity).

Table S5. Experimental IR, Raman, IINS and calculated DFT (B3LYP/6-311+G(d,p)) spectral data of compound **4** and its mono deuterated (OH → OD) derivative.

№	IR(OH), cm^{-1}	IR(OD), cm^{-1}	R(OH) cm^{-1}	R(OD) cm^{-1}	IINS(OH), cm^{-1}	IINS(OD), cm^{-1}	x DFT, cm^{-1}	Intensity [km/mol]	Potential energy distribution, %
1					21		23.37	0.5	τ(C7OO – C8OO) 91
2					41		39.54	0.09	τ(NO_2) 77; τ(CCC) 10; τ(C8OO) 8
3					53		69.69	3.03	τ(CCC) 41; γ(1-C7) 17; τ(C7OO + C8OO) 14
4					86				
5		122			106		97.72	1.3	τ(C7OO + C8OO) 50; ϱ(C1-C7 + C2-C8) 29
6	142,7				148	131	141.71	1.05	γ(C2-C8 + C4-N) 43; ϱ(C2-C8) 8
7		150,9		160 w	162	162	149.67	2.9	ϱ(C4-N + C1-C7 – C2-C8) 63; γ(C2-C8) 8
8	179,8			173	182	180	164.8	1.76	ϱ(C1-C7 + C2-C8) 65; ω(C7OO) 10
9		196		196	196		209.09	5.92	γ(C1-C7) 37; ϱ(C7OO) 18; γ(C4-N) 17
10			236			208			
11		244,4	244 sh	243	248	251			
12	263,7	263,7		261 w	270	273			
13	285,4				289		280.5	1.12	δ(CCC) 30; ν_{sym}(C1-C7 + C4-N) 21; ϱ(C4-N) 12; ϱ(C7OO – NO_2) 12
14	306,2		296	302	300		296.09	0.44	ϱ(C2-C8 – C1-C7 + C4-N) 36; ϱ(C8OO + NO_2) 26
15	307,1				309	306			
16		316,8		315		325			
17			326		331				
18	357,3	357,3	366	355	364	361	351.38	2.4	ν_{sym}(C2-C8 + C4-N) 33; δ(CCC) 22; δ(C8OO) 13
19		378,0	386	375 sh	388	382	379.44	0.17	τ(CCC) 35; γ(C2-C8 – C4-N) 26; ϱ(C7OO + C8OO) 20
20	403,0	401,1			408	408			
21					434				
22	432,0	439,7			438		415.24	7.51	τ(CCC) 57; γ(C2-C8) 14
23			446			446			

24	458,5		459 w		463		456.68	2.63	τ(CCC) 23; γ(C2-C8) 15
25		469,6				478			
26									
27	522,6	524,1	524	520	528	523	519.7	15.03	ϱ(NO_2 + C7OO) 53; ϱ(C4-N) 10
28		560,2	566	558			539.83	15.98	τ(CCC) 46; γ(C4-N) 17
29	575,7 sh				569	563	567.4	36.04	τ(C-O-H) 33; τ(CCC) 33
30	589,2	586,3	592		599	597	594.11	92.97	τ(C-O-H) 74
31		628,0 w					604.97	75.3	τ(C-O-H) 23; ϱ(C2-C8 + C4-N) 16; δ(CCC) 11
32		638,8 w							
33		653,8 w							
34	655		655	655	659		651.86	34.58	δ(C8OO) 42; δ(CCC) 29
35									
36	694,0	690 sh	689	684 w			669.95	15.67	δ_{asym}(C7OO – C8OO) 34; δ(CCC) 25
37	701,0	703,0	714 w		704	704 w	702.64	13.34	τ(CCC) 47; γ(C1-C7) 13
38	740,6	740,6	744	743			727.02	27.5	ω(NO_2) 53; τ(CCC) 18; γ(C4-N) 16
39		749,3 sh							
40	763,8		767		766	754	752.13	28.64	ν_{sym}(C1-C2 + C1-C7 + C2-C8) 26; δ_{sym}(C7OO + C8OO) 17; δ(CCC) 14
41		783,1	780				783.16	53.08	ω(C7OO + C8OO) 56; ϱ(C1-C7) 9
42	790,0	798,5	792	799	800		806.74	21.65	γ(C1-C7 + C2-C8) 30; ω(C8OO) 28
43	812,0	810,0	809	810 w		809	815.98	31.75	δ(NO_2) 40; δ(CCC) 19
44	835,0			827 w					
45		857,3							
46	862,2		861		870		873.23	27.74	γ(C-H) 70; τ(CCC) 9
47		870,2				880			
48	896,9								
49	910,4 w	913,3	913	911					
50	931,6	924,8 sh	928		938	921	920.13	26.21	ν_{asym}(C4-N – C2-C8) 32; δ(NO_2) 23; δ(CCC) 12

51							953.03	12.2	γ(C-H) 73; τ(CCC) 13
52	980,8 w	975 w	977 w		989	1001	999.47	0.35	γ(C-H) 84; τ(CCC) 12
53		995,2		994					
54		1013,6		1009 w					
55	1062,7	1065,0	1062	1064 w	1078	1084	1067.19	83.35	δ(CCC) 35; ν_{asym}(C8-O4 – C7-O2) 23; ν_{sym}(C3-C4 + C4-C5) 18
56	1120,6	1120,6 w	1120	1123			1118.49	145.27	ν(C7-O2) 40; ν_{sym}(C1-C2 + C1-C6) 13; ν(C8-O4) 7
57							1126.71	107.93	ν_{sym}(C3-C4 + C4-C5 + C5-C6) 24; ν(C4-N) 19; ν(C8-O4) 18; δ(CCC) 11
58		1140,8	1153						
59	1146,0	1150,5	1157	1158	1156	1155	1157.31	19.53	ϱ(C-H) 51; ν(C5-C6) 16; ν(C8-O4) 10
							1197.4	173.44	δ(C-O-H) 39; ν_{asym}(C1-C7 – C2-C8) 16; δ(CCC) 15
60	1236,3	1231,5	1249	1353 sh			1211.25	192.16	δ(C-O-H) 44; ν_{asym}(C1-C7 – C2-C3 + C2-C8) 23
61	1269,1	1299,0 br	1270			1275	1285.18	4.81	ϱ(C-H) 64; ν(C1-C6) 12
62	1309,6	1312,5							
63							1342.61	0.96	ν_{asym}(C1-C2 – C1-C6 – C2-C3 + C3-C4 – C4-C5 + C5-C6) 82
							1360.3	104.64	δ(C-O-H) 24; ν(C7-O2) 14; ν(C1-C7) 11
64	1351,0	1353,0	1353	1358			1365.7	300.9	ν_{sym}(ONO) 23; δ(C-O-H) 9; ν(C8-O4) 8
65	1388.0						1377.0	127.14	ν_{sym}(ONO) 42; ν(C4-N) 12; δ(NO_2) 11
66	1419,6			1406					
67	1430,2	1425,3	1425						
68	1439,0		1454				1432.9	10.95	ν_{asym}(C1-C6 – C2-C3 – C3-C4 + C5-C6) 44; ϱ(C-H) 18; ν(C2-C8) 7
69			1488						
70	1496,7	1491,9	1495	1491			1512.91	16.95	ϱ(C-H) 45; ν_{asym}(C1-C2 + C1-C6 – C3-C4 – C4-C5) 34; ν(C1-C7) 5
71	1536,0	1532,4	1522						
72			1543	1539					
73	1599,9	1590,3	1593	1591			1584.07	146.88	ν_{asym}(ONO) 66; ν_{sym}(C1-C2 + C4-C5) 14
74	1610,5	1610,5	1611	1614			1626.04	37.22	ν_{asym}(C1-C2 – C2-C3 + C4-C5 – C5-C6) 57
75			1630				1649.2	92.87	ν_{asym}(C1-C6 + C3-C4 – C4-C5) 42; ν_{asym}(ONO) 16

76				1649			
76	1683,0			1690			
77		1715,6 br		1713			
78				1724			
78	1732,0		1732		1799.68	324.15	ν(C8=O3) 77
79			1737 sh		1811.11	376.15	ν(C7=O1) 81
80	1952						
81		2066		2054			
82		2548		2249			
83		2637		2618			
84	2590		2620		3750.15	106.89	ν(O2-H) 100
85	2725		2698		3765.65	123.32	ν(O4-H) 100
86	2867		2864				
87			2974				
88				2958			
89	3091,0	3112	3087		3197.99	0.68	ν(C6-H) 94
90			3099	3097	3224.56	4.68	ν(C5-H) 94
91				3112	3238.32	12.44	ν(C3-H) 99

ν - stretching vibration; δ - scissoring vibration; ρ - rocking; ω - wagging; γ – out-of-plane; τ - twisting and torsional vibration; sh - shoulder and w – weak (small intensity).
